# Dendritic Cell-derived Extracellular Vesicles mediate Mesenchymal Stem/Stromal Cell recruitment

**DOI:** 10.1038/s41598-017-01809-x

**Published:** 2017-05-10

**Authors:** Andreia M. Silva, Maria I. Almeida, José H. Teixeira, André F. Maia, George A. Calin, Mário A. Barbosa, Susana G. Santos

**Affiliations:** 1 i3S – Instituto de Investigação e Inovação em Saúde, Porto, Portugal; 2 INEB – Instituto de Engenharia Biomédica, Universidade do Porto, Porto, Portugal; 3 Instituto de Ciências Biomédicas Abel Salazar, Universidade do Porto, Porto, Portugal; 4 IBMC – Instituto de Biologia Molecular e Celular, Universidade do Porto, Porto, Portugal; 5 Department of Experimental Therapeutics, The University of Texas MD Anderson Cancer Center, Houston, Texas USA; 6 Center for RNA Interference and Non-Coding RNAs, The University of Texas MD Anderson Cancer Center, Houston, Texas USA

## Abstract

Orchestration of bone repair processes requires crosstalk between different cell populations, including immune cells and mesenchymal stem/stromal cells (MSC). Extracellular vesicles (EV) as mediators of these interactions remain vastly unexplored. Here, we aimed to determine the mechanism of MSC recruitment by Dendritic Cells (DC), hypothesising that it would be mediated by EV. Primary human DC-secreted EV (DC-EV), isolated by ultracentrifugation, were characterized for their size, morphology and protein markers, indicating an enrichment in exosomes. DC-EV were readily internalized by human bone marrow-derived MSC, without impacting significantly their proliferation or influencing their osteogenic/chondrogenic differentiation. Importantly, DC-EV significantly and dose-dependently promoted MSC recruitment across a transwell system and enhanced MSC migration in a microfluidic chemotaxis assay. DC-EV content was analysed by chemokine array, indicating the presence of chemotactic mediators. Osteopontin and matrix metalloproteinase-9 were confirmed inside EV. In summary, DC-EV are naturally loaded with chemoattractants and can contribute to cell recruitment, thus inspiring the development of new tissue regeneration strategies.

## Introduction

Bone repair and regeneration requires a timely controlled inflammatory response^1^. An impaired pro-inflammatory response may compromise bone regeneration^2^, while excessive inflammation leads to increased bone destruction^3^. Resolution of inflammation during bone repair is dependent on the communication between immune cells and other cell populations in the bone microenvironment, including multipotent mesenchymal stromal/stem cells (MSC). Cell-to-cell communication may occur *via* direct contact or be mediated by cell-secreted factors, many of which likely carried by Extracellular Vesicles (EV).

Different EV populations are produced and released by cells, including apoptotic bodies, large microvesicles (200 nm–1 μm), and nanometric exosomes (30–200 nm), which carry proteins (e.g. cytokines) and nucleic acids (DNA, mRNA, microRNA) capable of modulating the activity of target cells^4^. Exosomes, that originate in multivesicular bodies inside the cells, are actively loaded and secreted^5^, and show some degree of cell targeting^6, 7^. They are secreted by virtually all cells, and can be found in biofluids. Therefore, exosomes may act in locations distant from those where they were produced and released^8^. EV have ascribed functions both in homeostasis and pathological conditions^9^, being most studied in the cancer field, for their potential use in cancer therapy^10^, and as immune mediators^9^. Thus, EV likely also impact the contribution of immune cells to tissue repair processes^9, 11^. As part of their immumodulatory activity, DC exosomes were shown to promote granulocyte migration, containing enzymes that participate in synthesis of chemotactic molecules^12^. *In vitro* and *in vivo* studies suggest beneficial roles for EV in tissue repair^13, 14^, likely through inflammation modulation.

MSC have been intensively explored for their potential use in stem cell therapies for tissue repair and regeneration, including in several ongoing clinical trials^15^. They are particularly interesting for bone tissue regeneration due to their immunomodulatory properties, potential to differentiate along osteogenic and chondrogenic lineages, and supportive role for other cells in the microenvironment^13^. MSC have been shown to home into locations of active inflammation^16^. However, cell mobilization and retention at injury locations is usually ineffective. Thus enhancing endogenous or transplanted cell recruitment and engraftment could improve current MSC-based therapies.

Our previous work showed that DC promote MSC migration *in vitro*, through the secretion of paracrine factors^17^. Other studies demonstrated the same capacity for other immune cells, such as macrophages, NK cells and T cells^18^. Also, monocyte/macrophages and their secreted EV have been shown to enhance MSC osteogenic differentiation^19^.

Thus, in the current work we hypothesized that the paracrine chemoattractant effect of DC upon MSC was largely mediated by DC-secreted EV. To test our hypothesis, we used EV secreted by primary human DC and investigated their biologic effect on MSC. The results presented here confirm our hypothesis, showing that DC-derived EV were internalized by MSC, and promoted their migration in a dose-dependent manner. Interestingly, DC-derived EV did not impact neither MSC proliferation nor osteogenic differentiation. The molecular composition of EV revealed that they were enriched in molecules involved in cell recruitment, like osteopontin, and also contain the matrix metalloproteinase (MMP)-9.

## Results

### EV isolated from Dendritic Cells are enriched in exosomes

**Figure 1:**
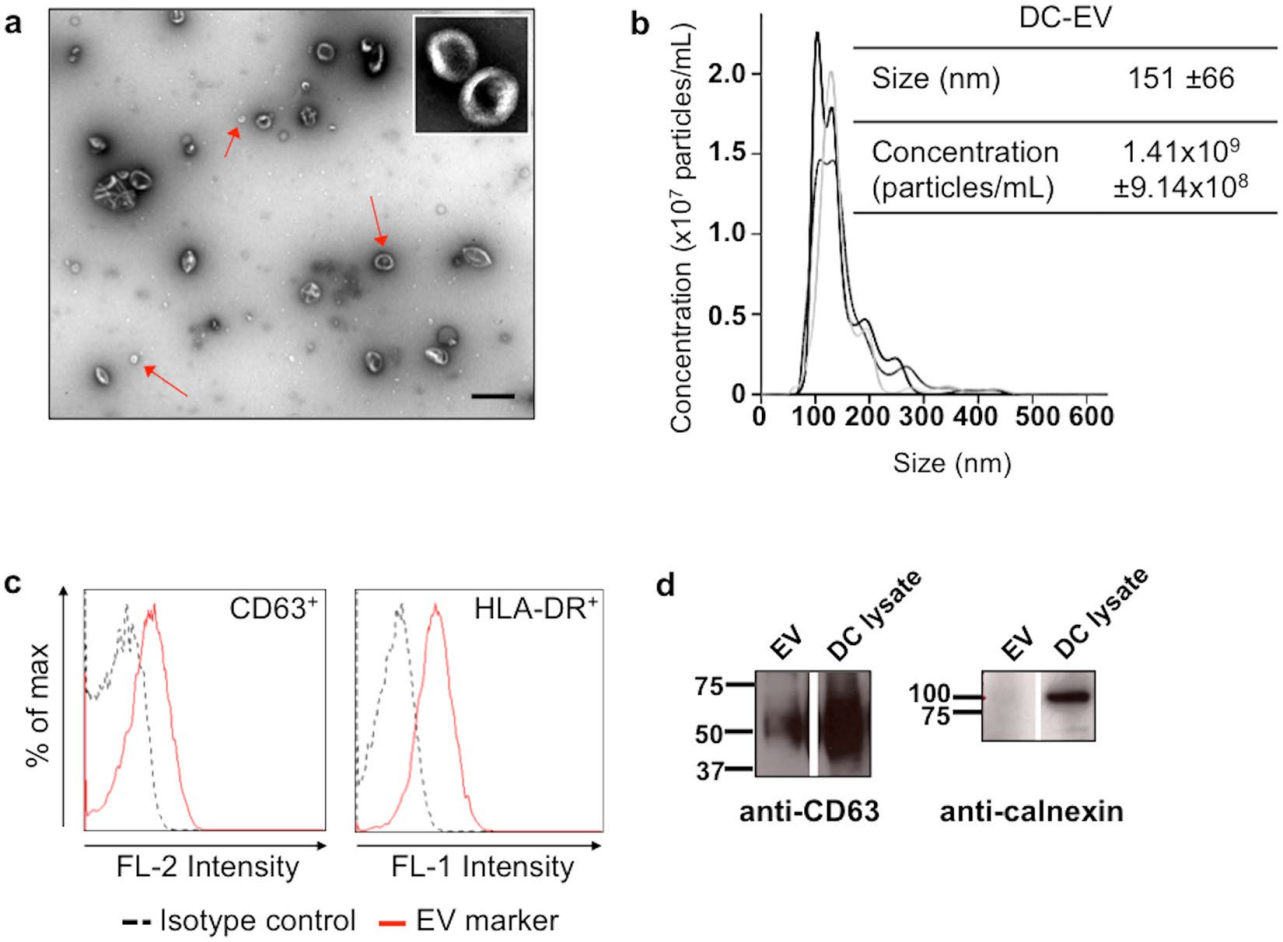
Characterization of exosome-enriched EV isolated from human monocyte-derived dendritic cells cultures. (**a**) Morphology of the isolated EV as imaged by transmission electron microscopy. Red arrows point to some EV of different sizes (scale bar: 500 nm). Image inset shows imaged EV in finer detail, at a magnification of 100,000x. (**b**) Characterization of concentration versus size distribution of isolated EV, by nanoparticle tracking analysis. Plot is representative of EV size and concentration analysis with NanoSight NS300. (**c**) Representative plots of conventional flow cytometry for the identification of the exosomal marker CD63 and the antigen-presenting cell marker HLA-DR on DC-derived EV, after immunostaining of DC-EV coupled to beads with CD63-PE and HLA-DR-FITC antibodies. (**d**) Representative western blots for the detection, as indicated, of the exosomal marker CD63 (non-reducing conditions) in isolated EV and DC whole lysates, and the endoplasmic reticulum marker calnexin (reducing conditions). Full gel scans are available in Supplementary Fig. S5. For all techniques n ≥ 3.

In order to isolate a population of EV enriched in exosomes, supernatants from primary human monocyte-derived DC, cultured with EV-depleted FBS, were processed via the classical differential (ultra)-centrifugation method. EV obtained were characterized for vesicle morphology and presence/absence of characteristic membrane protein markers (Fig. 1). For each EV preparation the amount of protein was quantified, and protein concentration was used as a measure for the amount of EV to be used in all the functional assays. TEM analysis revealed the presence of vesicles with a lipid bilayer and cup-like morphology. Vesicles sub-populations with different sizes could be distinguished, all of them with a diameter under 200 nm (Fig. 1a). For an accurate analysis of size distribution, EV suspensions were analysed by NTA. Results obtained show EV with an average diameter in solution of ~150 nm, and including subpopulations of different sizes (Fig. 1b). Similar results were obtained by DLS, with a polydispersity index of 0.36 ± 0.06 for EV suspensions. In terms of protein markers, both western blotting and flow cytometry results indicate that the EV population isolated contained CD63 (Fig. 1c,d). The antigen presenting cell marker HLA-DR was also detected at high levels on isolated EV, in agreement with their DC origin (Fig. 1c), while the endoplasmic reticulum marker calnexin was absent from EV (Fig. 1d). Overall, our data indicate that the EV population isolated was highly enriched in exosomes.

### DC-derived EV are internalized by MSC without negatively impacting MSC proliferation or differentiation

The most ascribed functions of DC-derived EV (DC-EV) are related to immune responses. Here we investigated their potential role in linking inflammation with repair, by interacting with MSC and influencing their behaviour.

**Figure 2:**
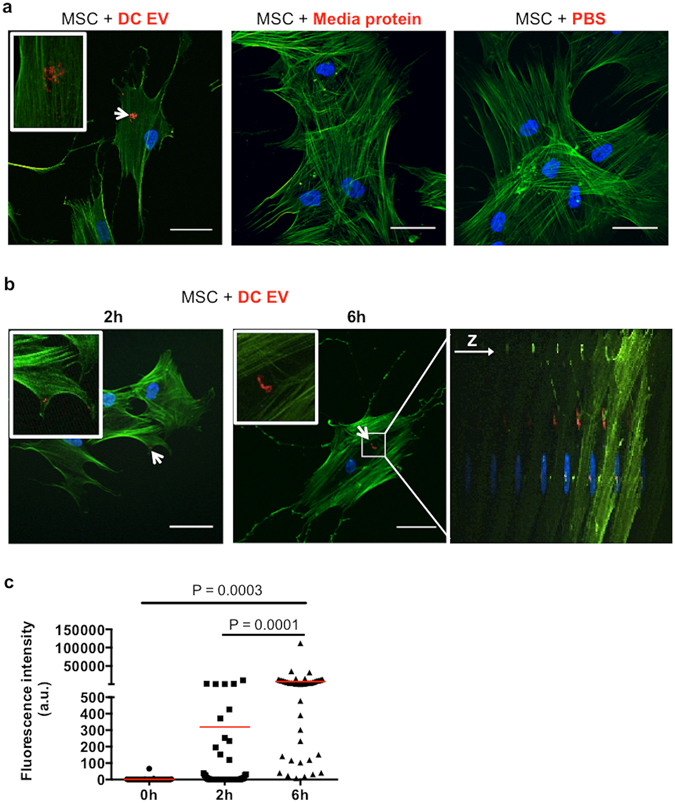
DC-derived EV are internalized by MSC. DC-derived EV were labelled with the lipophilic dye PKH26 before being co-incubated with MSC. Cells were labelled for actin cytoskeleton in green and nuclei in blue. (**a**) MSC with internalized red-labelled EV after 6 h of co-incubation (left panel). Specificity of EV labelling and internalization were accessed adding to MSC protein pellets of ultracentrifuged cell-free media (middle panel) or PBS (right panel) red-labelled as for EV, with no red staining detected. (**b**) MSC with internalized red-labelled EV after 2 h (left panel) and 6 h (middle panel) of co-incubation. Right panel shows the z-axis of an image stack, at 6 h. White arrows point to internalized EV, which are highlighted in picture insets. Z-stacks of images were acquired by laser scanning confocal microscopy and processed on Fiji software. Scale bar: 50 μm. (**c**) Quantification of DC-EV internalization by measuring the fluorescence levels (a.u. = arbitrary units) of the red channel in cell ROIs previously defined according to phalloidin-AF488 staining. Red dash represents the mean and p-values are indicated as determined by 2-way ANOVA, followed by uncorrected Fisher’s multiple comparisons testing.

First we examined the capacity of DC-EV to interact with MSC, by labelling them with the lipophilic dye PKH26 before co-culturing with MSC. Upon incubation with cells, labelled DC-EV could be detected inside MSC, suggesting their internalization (Fig. 2a, left panel). Importantly, to preclude that PKH26 micelles or other form of labelled structure could lead to artefacts in internalization detection, both protein pellets from ultracentrifuged cell-free media and PBS (EV vehicle) were labelled in the same way and incubated with MSC, leading to no detectable staining inside the cells, even upon 6 h of incubation (Fig. 2a, right panel). Following the kinetics of DC-EV uptake, after 2 h of co-incubation, internalized DC-EV start to be detected inside MSC, in small scattered dots, close to the cell membrane (Fig. 2b, left panel). After 6 h of incubation, abundant DC-EV could be detected inside MSC, mostly clustered and well inside the cell cytoplasm, as evidenced by the z-direction panel (Fig. 2b, right panel). The amount of fluorescence detected inside cells was quantified, showing a significant increase in the levels of fluorescence for internalized EV at 6 h of co-incubation, when compared to control and to 2 h of co-incubation (Fig. 2c).

**Figure 3:**
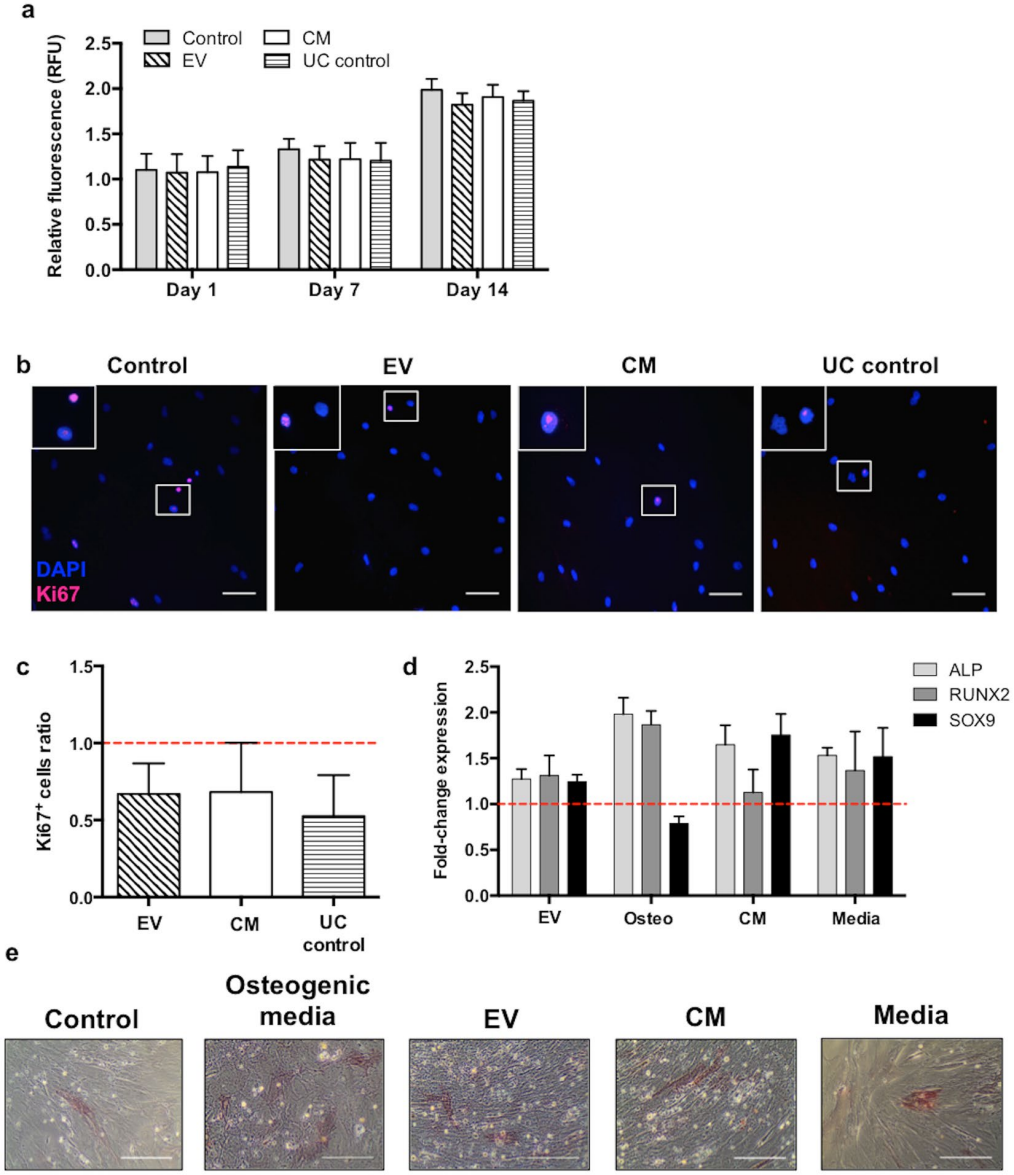
Stimulation of MSC with DC-derived EV is not cytotoxic and do not affect significantly their proliferation nor differentiation capacity. (**a**) MSC were incubated alone, with DC-derived EV (10 μg of protein), total conditioned media or 100 K ultracentrifugation supernatant (UC control) for 14 days. At days 1, 7 and 14, MSC were incubated with resazurin and conversion to resorufin was determined by fluorescence measurement (RFU = relative fluorescence units). Relative fluorescence at each timepoint normalized to day 0 is depicted. (**b**) MSC were incubated alone (Control), with 50 μg protein from EV, total conditioned media (CM), or 100 K ultracentrifugation supernatant (UC control) for 24 h, before staining for the Ki67(-AlexaFluor 647; pink) proliferation marker and DAPI for nuclei (blue). Scale bar: 100 μm. (**b**) Stained cells were imaged by fluorescence microscopy in 20 random fields, the total number and the number of Ki67^+^ cells were counted, and are represented as a ratio to non-stimulated control condition (dashed line) (n = 4 different DC donors). All bar graphs represent average ± SD. n = 3 different DC donors. (**d**) Fold-change gene expression by RT-qPCR (average ± SD) of osteogenic markers (ALP and RUNX2) and a chondrogenic marker (SOX9) of MSC stimulated with DC-derived EV, osteogenic media (osteo: supplemented with Dexamethasone 10^−7^ M, β-glycerophosphate 10^−2^ M and ascorbic acid 5 × 10^−5^ M), conditioned media (CM), or protein extracted from complete media (including cytokines) that was not in contact with cells (media), comparatively to MSC in basal conditions (dashed line; n = 2 different DC donors). (**e**) ALP activity staining (pink/red), associated with early osteogenic differentiation, of MSC cultured in the presence of the indicated stimuli for 14 days. Although ALP activity staining is similar in stimulated conditions, only osteogenic media lead to morphological changes of MSC characteristic their osteogenic commitment. Scale bar: 1 mm.

To determine if DC-EV had an impact on MSC metabolism, we exposed the cells to EV, total conditioned media (CM) or the EV-depleted 100 K supernatant (UC control), and monitored their metabolic activity for up to 14 days. The results obtained show no significant differences in MSC metabolic activity, in presence of DC-EV or the 100 K supernatant (Fig. 3a). To further confirm these results, we evaluated MSC proliferation using the Ki67 marker. Results are illustrated in Fig. 3b and show dividing cells labelled for Ki67 in the nucleus. Quantification of proliferating MSC across different experiments showed no significant reduction of MSC proliferation in presence of DC-EV (Fig. 3c).

Next, we tested if DC-EV influenced MSC fate decision, inducing higher osteogenic or chondrogenic differentiation. At the gene expression level, results obtained indicate that EV stimulation promoted a slight increase in the expression of ALP and RUNX2 genes (osteogenic lineage markers), and also SOX9 (chondrogenic lineage marker), relatively to unstimulated MSC, at 6 h after stimulation (Fig. 3d). However, this increase was non-significant and below 1.5 times, much lower than promoted by osteogenic stimulation. Moreover, DC conditioned media, and even EV-producing media that was never in contact with cells (Media; contains EV-depleted FBS and DC-differentiating cytokines), promoted a higher increase in ALP and RUNX2 expression (Fig. 3d). To clarify these results, we cultured MSC in presence of the different stimuli for 14 days and looked at the ALP activity staining. Representative images are shown in Fig. 3e and confirm the results obtained by gene expression, with more ALP activity in presence of DC-EV than in basal conditions, but less than osteogenic stimulation. Also here DC conditioned media was capable of stimulating ALP activity to comparable levels as DC-EV (Fig. 3e). Together, these results indicate that any factors that may interfere with MSC osteogenic differentiation were not preferentially associated with DC-EV.

### DC-derived EV promote MSC recruitment and migration

**Figure 4:**
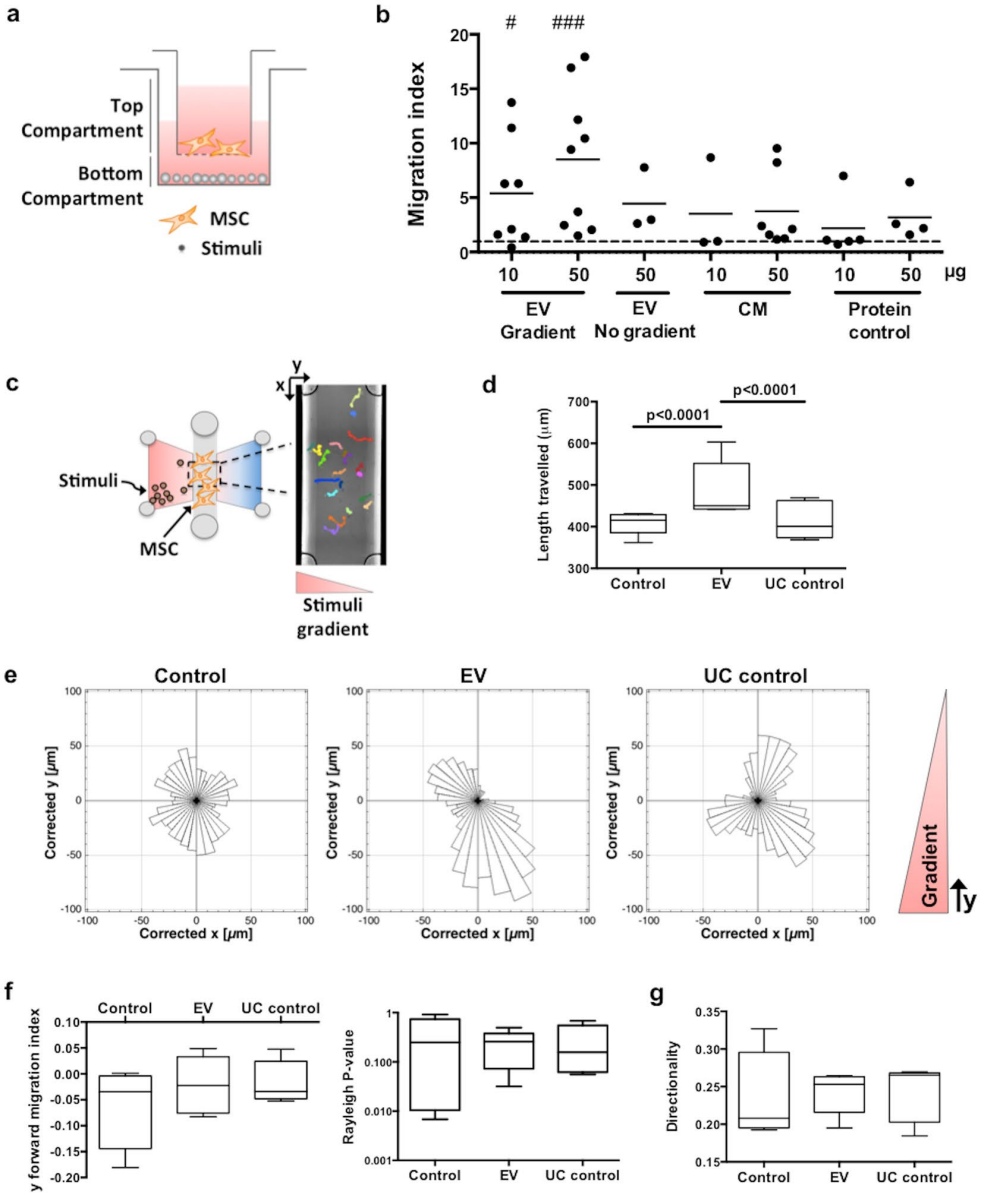
DC-derived EV specifically promote MSC migration. (**a**) Representation of the transwell system set up for MSC recruitment assay. EV or control stimuli were added to the bottom compartment, acting as chemoattractants for MSC seeded inside the gelatin-coated transwell insert. For gradient disruption, 50 μg EV were diluted in media that was then equally distributed in the top and bottom compartments (no gradient). (**b**) MSC were allowed to migrate for 8 h, and then stained with DAPI. Total number of migrated cells adhered to the outer surface of the insert membrane were counted and migration index calculated to non-stimulated control. The dashed line represents the migration index of non-stimulated control. Each dot represents an independent experiment (n = 3 to 9 DC donors) and the mean value of cells migrated is indicated as a dash. For statistical analysis Kruskal-Wallis test, followed by uncorrected Dunn’s multiple comparison test was used, ^#^P = 0.0152 and ^###^P = 0.0002, compared to non-stimulated control. (**c**) Representation of the chamber setup for MSC chemotaxis assay. Stimuli were added on the left side of the chamber and a gradient was formed across the central channel, where MSC were seeded (n = 5 DC donors). For each condition, MSC were followed for 13 h by time-lapse video microscopy, and cell pathways were then determined by manual tracking, as represented by the coloured tracks overlay on the exemplificative central channel image. See also Supplementary Fig. S1. (**d**) Box plots represent total length of the pathways travelled by MSC. Statistical analysis used 2-way ANOVA, followed by uncorrected Fisher’s multiple comparison test, p values are indicated. (**e**) Representative rose diagram of MSC direction of migration (x and y position normalized for cell’s initial position). (**f**) MSC forward migration index in gradient y-direction (left panel), and p-value of cell direction uniformity as determined by Rayleigh test (right panel). (**g**) Directionality of MSC migration. All box plots are min to max, with median value indicated.

Since EV are secreted biological messages that can travel in body fluids, it is likely that they may participate in chemotactic processes and exert their function on cells located far from their origin. So, in order to investigate their potential to recruit MSC, different amounts of DC-EV were placed in the bottom of tissue culture wells and transwell inserts with seeded MSC were placed on top (Fig. 4a). This setup allows the establishment of an EV gradient from the bottom to the top compartment. The number of MSC that migrated in response to DC-EV stimuli or controls were quantified across 9 different DC donors. The results are presented as migration index relative to control, and clearly show that DC-EV were able to stimulate MSC recruitment in a dose dependent manner (Fig. 4b). A statistically significant increase in MSC migration was observed for as little as 10 μg of EV, when compared with control conditions (Fig. 4b). On the other hand, the same amount of protein from total DC conditioned media (CM) or EV-producing media that had not been in contact with DC (containing EV-depleted FBS and DC-differentiating cytokines), did not recruit significantly more MSC than the negative control (Fig. 4b), further confirming the specificity of MSC recruitment by DC-EV. As a control for the effect of gradient on MSC migration, 50 μg of EV were equally distributed on the top and bottom compartments of the transwell migration system (EV No gradient), disrupting the chemotactic EV gradient. In these conditions, MSC migration was similar to CM control conditions, and lower than in the presence of a gradient, for the same amount of EV (50 μg, EV Gradient), indicating some chemotactic role for EV on MSC migration.

To further validate the capacity of EV to promote MSC migration, 10 μg of DC-EV were added to a microfluidic chamber, and the migration pattern of MSC seeded across the EV gradient was followed by time-lapse video microscopy (Fig. 4c, Supplementary Fig. S1 and Supplementary Video S1). Different parameters of cell migration were then determined by image analysis (Fig. 4d–g). Under the influence of 10 μg of EV gradient, the length of the cell migration pathways was statistically significantly higher than for UC or negative control (Fig. 4d). This result confirmed the previous transwell assay results on the capacity of DC-EV to specifically promote MSC migration, in doses as low as 10 μg.

Analysing the directionally of MSC migration in response to the gradient, despite the tendency for MSC to migrate in direction of EV (y coordinates < 0) comparing to control, or 100 K supernatant (UC control) (Fig. 4e), there were no statistically significant differences for this DC-EV concentration, as quantitatively assessed by the forward migration index across the y axis (yFMI; axis of gradient establishment), and the p-value of the Rayleigh test for uniformity of the pathways followed by MSC (Fig. 4f). Similarly, while directionality of MSC migration was slightly higher for conditions with stimuli (EV and UC control), it was not significantly different from control conditions (Fig. 4g).

### DC-derived EV are rich in mediators that can promote cell migration

**Figure 5:**
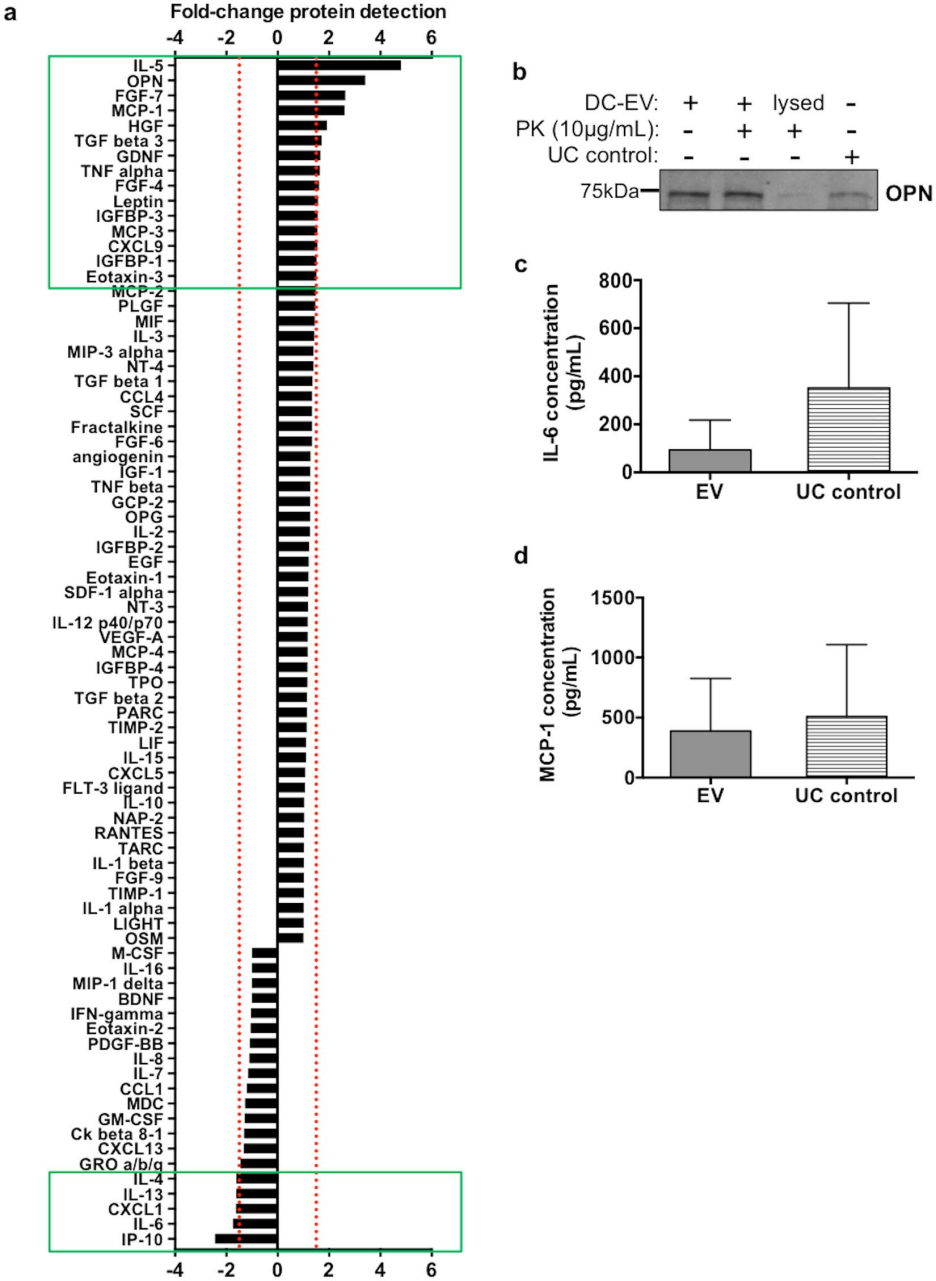
DC-derived EV are enriched in mediators with reported chemoattractant properties. (**a**) Chemokines and growth factors enriched/depleted in DC-EV comparatively to DC conditioned media, as assayed by antibody array. Red dashed lines represent the fold-change thresholds of 1.5 and −1.5. Mediators with an absolute fold-change ≥1.5 are highlighted by the green box. See also Supplementary Fig. S2. (**b**) Detection of osteopontin (OPN) in the DC-derived EV fraction (DC-EV) and 100 K supernatant (UC control) by western blot. Proteinase K (PK) digestion, before or after vesicle lysis, were performed as indicated. Full gel scan is available in Supplementary Fig. S5. Relative concentration (average ± SD) of (**c**) IL-6 and (**d**) MCP-1 in DC-derived EV (after proteinase K digestion) and 100 K supernatant (UC control), as determined by ELISA. For all assays, EV, 100 K supernatant and conditioned media from 3 different DC donors were pooled together.

Considering the chemotactic action of DC-EV on MSC, we then investigated their protein content. We focused on the analysis of cytokines, chemokines and metalloproteinases, as they are some of the most important modulators of cell migration. An antibody array was used to screen the content of DC-EV. Total conditioned media was used for comparison, in order to obtain the molecules that are enriched inside EV (Supplementary Fig. S2 shows an original array image). The results obtained are illustrated in Fig. 5a, and indicate that some mediators are much enriched and others much depleted in DC-EV. Interestingly, IL-5, osteopontin (OPN), FGF-7 and MCP-1 appeared highly enriched in EV (fold-change >2), while IP-10, IL-6, CXCL1 and IL-4 were depleted from EV preparations (fold-change <−1.5).

The array results were validated by alternative approaches for chemokines selected from those most differently enriched or depleted in DC-EV (Fig. 5b–d). When probing DC-EV for osteopontin the results obtained confirm the array data, showing an enrichment in EV, relative to the 100 K supernatant (UC control). Moreover, the results indicate that osteopontin was present inside DC-EV, as it was detectable even after EV treatment with proteinase K (Fig. 5b). Importantly, when EV were lysed before proteinase treatment, osteopontin was not detectable, as it is no longer protected from degradation (Fig. 5b). ELISA assays confirmed that IL-6 was highly depleted from DC-EV, in comparison with the 100 K supernatant (Fig. 5c). The enrichment of MCP-1 in EV, at least relatively to the 100 K supernatant, could not be confirmed (Fig. 5d). The observed similar concentration of MCP-1 in EV and 100 K supernatant (UC control) suggests that it may be secreted in both fractions, *i.e*. as a soluble mediator, and associated with EV.

**Figure 6:**
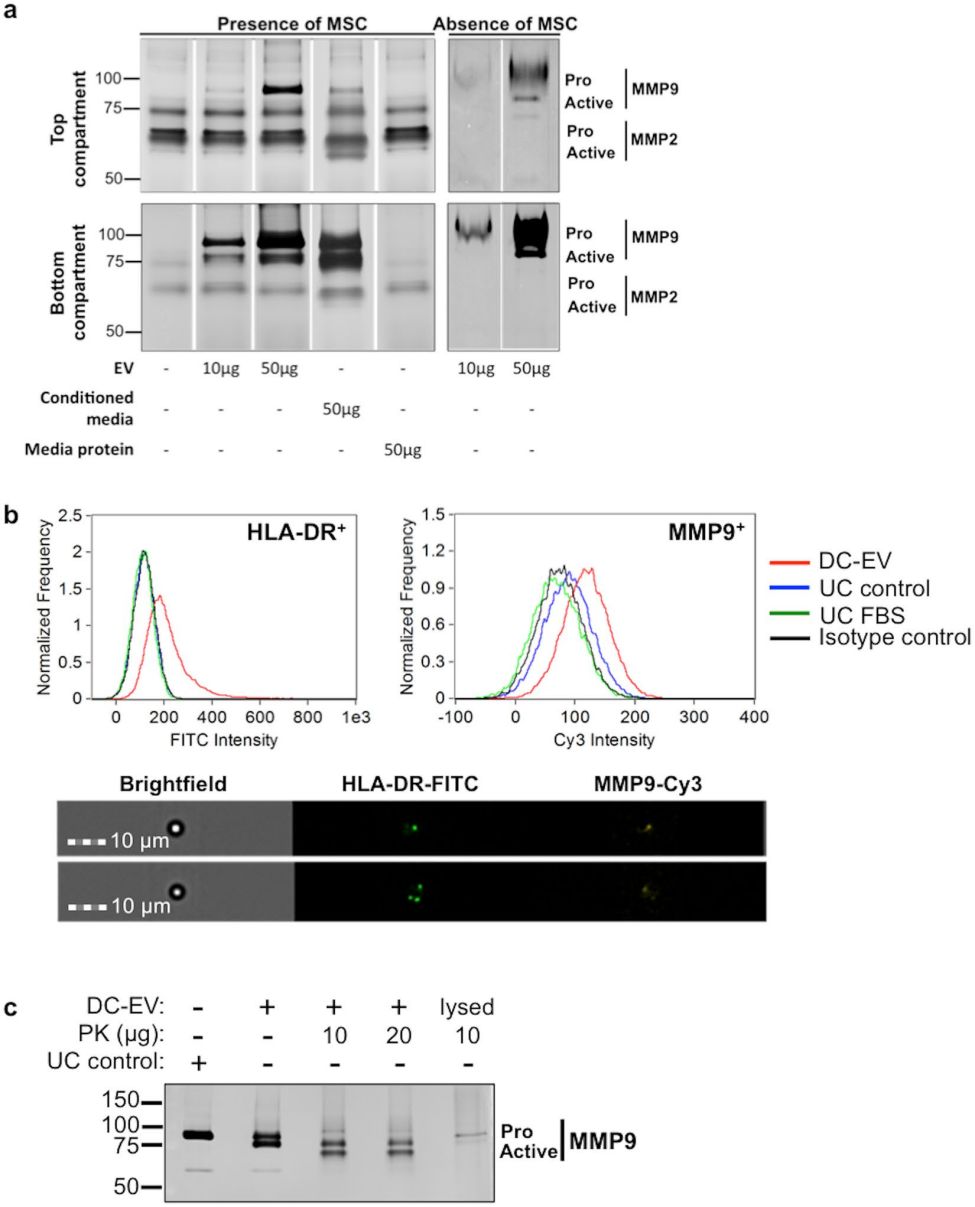
DC-derived EV contain MMP-9 capable of promoting MSC migration. (**a**) Detection of MMP-9 and MMP-2 activity by gelatin zymography in media collected from transwell assays. Cell culture media from top and bottom compartments of MSC recruitment transwell assays was collected separately. Protein was precipitated from these media (Presence of MSC) and from DC-EV pellets (Absence of MSC), resolved by gelatin zymography, and proteolytic activity was detected as clear bands on Coomassie Brilliant Blue stained gels. Bands corresponding to the proenzyme and the active form of each MMP are indicated at the corresponding molecular weight next to gels images. (**b**) Representative imaging flow cytometry plots (top panel) and images (bottom panel) of MMP-9 and HLA-DR detection in DC-derived EV coupled to beads. Beads with EV were stained with HLA-DR-FITC antibodies before being permeabilized and stained using anti-MMP-9 followed by Cy3-secondary antibodies, being detected as defined dots in the periphery of the beads. (**c**) Gelatin zymography detection of MMP-9 in DC-derived EV (DC-EV), and the 100 K ultracentrifugation supernatant control (UC control). Proteinase K (PK) digestion, with or without previous vesicle lysis, were performed as indicated. Pro-MMP-9 and active MMP-9 are indicated at the corresponding molecular weight. Full gel scans are available in Supplementary Fig. S6.

Our previous results indicated that MMP-9 was produced abundantly by DC and could be involved in MSC recruitment by these cells ^17^. Thus, we first determined if MMP-9 and MMP-2, the two most common gelatinases, were present in media collected from the transwell migration assays described in the previous section (Fig. 4a and b). As shown in Fig. 6a, when DC-EV were added to the bottom compartment, a clear increase in the levels of both proenzyme and active MMP-9 was detected. This pattern was replicated in media from the top compartment, but with lower amounts of MMP-9, as expected. MMP-9 could also be found in total DC conditioned media, but not in media that had not been in contact with these cells. On the other hand, MMP-2 was only detected when MSC were present, mainly in the top compartment and at relatively constant levels in all conditions, indicating that it is likely produced by MSC.

Knowing that MMP-9 was present in the EV fraction we then proceeded to investigate if MMP-9 was inside EV, by two independent approaches. First, we stained for MMP-9 and detected it by imaging flow cytometry (Fig. 6b, bottom panel), in a pool of permeabilized DC-EV coupled to latex beads. A representative cytometry profile is illustrated in Fig. 6b (top panel) and shows that DC-EV stain for MMP-9 at higher levels than isotope controls or MMP-9 stained beads that had been incubated with the 100 K supernatant (UC control), which indicates specific MMP-9 staining. However, this could not preclude that MMP-9 could pellet in the ultracentrifugation, or be associated with the surface of EV. So, in order to clarify if MMP-9 was indeed inside the vesicles, gelatin zymography of undigested versus proteinase K-digested EV was performed. Results are illustrated in Fig. 6c and show that active form of MMP-9 was present in EV, and still detectable after proteinase K digestion, but was no longer detectable when EV were disrupted before proteinase digestion. Interestingly, the pro-MMP-9 form, most abundant in 100 K supernatant, but also detectable in the EV fraction, was not resistant to proteinase K digestion, indicating that it may be extraexosomal, or be associated on the outer side of EV.

Together these results show that DC-EV are enriched in chemotactic mediators and MMP-9, all likely contributors to MSC migration.

## Discussion

In the current study we investigated for the first time the influence of DC-EV on different aspects of MSC biology, in particular their migration, and related that effect with EV content.

In order to improve the regenerative processes there is a need to enhance our understanding on the coordination of the different stages of repair. In particular the signals that contribute to the articulation between inflammatory and repair phases are crucial, as reduced or exacerbated inflammatory responses can be profoundly deleterious to repair^20^.

Cells can secrete a variety of EV with different functions, namely exosomes, microvesicles and apoptotic bodies, distinguished by their size, composition, biogenesis pathway and function^4^. So, we first characterized the population of EVs isolated by differential (ultra)centrifugation from DC cultures, according to the minimal criteria parameters recommended by the International Society of Extracellular Vesicles, in order to confirm their enrichment in exosomes^21^. Overall, morphology and size distribution of the isolated vesicles, the presence of the endosomal protein marker CD63, and the absence of a protein marker characteristic of the endoplasmic reticulum (calnexin), indicate that our methodology allows, according to these criteria, the isolation of vesicle populations enriched in exosomes of endosomal origin. Moreover, although CD63 has been described in other sub-populations of DC-EV in a very recent study^22^, the EV population isolated here is similar to the one described by those authors as the “100 k pellet”, both in terms of NTA profile and protein markers.

The mechanism of EV action likely depends on the way they interact with target cells. The main forms of interaction reported are EV-mediated activation of cell surface receptors, triggering signal transduction pathways; fusion with the cell membrane, releasing EV content to the cell cytoplasm; or internalization, delivering EV content already inside the cell^23^. Although other mechanisms of interaction cannot be ruled out, our results clearly show that MSC internalize DC-EV, which are found in clusters, well inside the cytoplasm of the cells. This vesicle distribution upon internalization was previously reported^19, 24^, suggesting an active endocytosis of the vesicles, that are incorporated in different compartments of the endocytosis pathway along MSC cytoplasm, and consequently lead to an active processing of the molecules they carry^23^. Despite the accumulation of DC-EV inside MSC no deleterious effects were observed in their metabolism, capacity to proliferate or differentiate towards osteogenic or chondrogenic lineages.

At the functional level, DC-EV appear to specifically promote MSC recruitment, as they did not impact significantly other aspects of MSC biology, such as their proliferation or differentiation. This action somehow correlates with the biological role of DC in immunological surveillance, and their transient presence at injury locations. On the other hand, recent reports suggest that MSC-derived EV can promote their own osteogenic differentiation^25–29^. Also, EV derived from monocytes/macrophages and platelet lysates, were reported to stimulate MSC osteogenic differentiation^19, 24^, while osteoclasts secrete EV containing microRNA that inhibit MSC osteoblastic differentiation^30^.

Of note, we had previously shown that DC interact with MSC, promoting their recruitment trough paracrine factors^17^. In this work, we demonstrate for the first time that the paracrine MSC migration by DC is mainly mediated by a functional exosome-enriched EV population secreted by DC. The EV-depleted 100 K supernatant was used to account for possible effects due to soluble proteins secreted by DC that could co-precipitate with isolated EV. Indeed, MSC migration increases with increasing quantities of chemoattractant DC-EV, which work even in the absence of the EV producing cells. A similar pattern of MSC migration was previously reported for comparable quantities of exosomes (5 μg and 50 μg) derived from human platelet lysates^24^, but DC-derived EV appear able to promote higher recruitment of MSC across the transwell membrane. Another recent study indicates that iPS-derived MSC produce EV capable of promoting BM-MSC migration, in a wound closure assay^31^. The authors also report that iPS-MSC-derived EV enhance MSC differentiation and proliferation^31^. Importantly, DC-EV effect on MSC migration was specific for the isolated EV, and migration was not significantly promoted by the same amount of protein from any of the controls tested: (i) total conditioned media, which contains both EV and soluble mediators; (ii) ultracentrifuged protein pellets derived from cell-free EV-producing media, which contains FBS proteins (although EV depleted) and DC differentiating cytokines; or (iii) ultracentrifugation supernatant, which contains the soluble proteins secreted by DC but is depleted from EV. Furthermore, this effect was observed using two different assays, several independent DC donors and two independent MSC donors, supporting an overall new mechanism of MSC migration modulation by DC-derived EV.

Cell migration may be affected by changes to either their chemokinesis, the movement of cells without a defined direction due to the presence of a stimuli, and/or their chemotaxis, the directed movement of cells towards a given stimuli^32^. In previous reports, cell-secreted chemical mediators were shown to specifically induce higher chemokinesis on their target cell^33, 34^. On the other hand, a recent study shows that exosomes play a crucial role in directional cell movement^35^. Although we could observe a tendency for MSC directional movement towards DC-EV, it did not reach statistical significance, likely due to the sensitivity of the assay for the gradient that was established with the small concentration of EV used (10 μg). In addition, the presence of an EV-directing gradient appeared to be important to promote MSC migration, since an equal amount of DC-EV without a gradient was not capable of significantly promoting MSC migration in the transwell system. Together, our results indicate that the presence of EV likely has effects on both MSC chemotaxis and chemokinesis. Considering the reported capacity of MSC to migrate to injury sites^36^, these effects of DC-EV could be important for MSC mobilization in a clinical context.

In order to further understand the mechanisms underlying the observed effect on MSC recruitment and identify candidate molecular mediators, we then explored the content of DC exosomes. Among the mediators mostly enriched in DC exosomes, OPN and MCP-1 were identified. The enrichment of OPN inside DC-derived EV was confirmed by Western Blot, which also showed that proteinase K was not able to degrade OPN, while it was protected by EV. Importantly, the secretion of OPN greatly increases in injury situations, and different studies suggest its capacity to promote MSC migration. Indeed, similar quantities of recombinant OPN were shown to promote human MSC migration in chemotaxis chambers to a level just slightly lower than in our study^37^, an effect also demonstrated in rat bone marrow-derived MSC^38^. In addition, some studies have described intracellular roles for OPN. An early study with foetal rat calvaria stromal cells found an association between increased cell migration and increased levels of intracellular OPN, which accumulated close to the cell membrane, together with CD44^39^. These findings were later confirmed for murine osteoclasts^40^ and macrophages^41^. Thus, it is possible that OPN inside DC-EV may modulate MSC migration upon EV internalization, without the need for its binding to a receptor on MSC surface.

On the other hand, MCP-1 enrichment in DC-derived EV comparing to ultracentrifugation supernatant could not be confirmed by ELISA, as it was found in both at similar levels. Nonetheless MCP-1 appeared to be carried inside the vesicles or may be associated on their surface. Contradictory roles for MCP-1 effects on human MSC migration have been reported. Dwyer *et al*. demonstrated a dose-dependent increased migration of iliac crest-derived MSC in the presence of recombinant MCP-1 and conditioned media from breast tumor cells, which was rich in this chemokine^42^. On the other hand, Ringe *et al*. stated that MCP-1 had no chemotactic effect on iliac crest-derived MSC. However, among the three MSC donors those authors tested, one does not express CCR2 (MCP-1 receptor), and the other two show migration in the absence of a chemoattractant to be the highest among all the experimental conditions^43^. Most interestingly, this chemokine was also demonstrated to promote murine adult bone marrow-derived MSC migration into the heart of transgenic mice specifically overexpressing MCP-1 in this organ, whereas only trace amounts of cells were found in other organs^44^. This work also reported HGF as a potent MSC chemoattractant, a growth factor also enriched in the DC-derived EV reported here.

Of note, SDF-1 was not found at high levels inside DC-derived EV. This chemokine is often considered the gold standard mediator of stem cell migration, including MSC, and in particular in injury pro-inflammatory environments^45, 46^, and thus our results support the relevance of alternative mediators for MSC migration. Also cytokines, such as IL-6, and IL-4 and GM-CSF (both added exogenously for DC differentiation) were found to be depleted inside EV, thus providing an important control for EV purity and specificity of action.

In addition to chemoattractant mediators, we also studied the contribution of metalloproteinases, namely gelatinases, to MSC migration in our *in vitro* model. MMPs are a family of secreted enzymes that are described to promote cell migration and invasion via degradation and remodelling of extracellular matrix components. However, they can potentially also have intracellular activity, as they are able to cleave several intracellular proteins, including cytoskeletal proteins^47^, although the functional outcome of such processes is not yet completely uncovered. Our previous results suggested a role for MMP-2 and MMP-9 in MSC recruitment by DC^17^. In agreement with those results, we found an increase in MMP-9, namely pro-MMP-9, in media of the transwell migration experiments, when DC-derived EV were present, and detectable MMP-2 only when MSC were present. However, in this setup we could not confirm the cell origin of MMPs, since MSC secrete higher levels of MMPs upon stimulation with different cytokines^48^. Thus, we further tested the presence of MMP-9 inside DC-derived EV. The presence of MMPs in EV, namely MMP-2 and MMP-9, has been previously described for several cell populations, including neutrophils^49^ and MSC^50^. Our results indicate that the EV fraction is positive for MMP-9, as detected by flow cytometry. Moreover, Western blot analysis confirmed that active forms of MMP-9 were found inside EV, as they were resistant to proteinase K digestion, while pro-MMP-9 was likely mainly extraexosomal, either soluble or associated with vesicles membrane. Thus, EV contain functional MMP-9 that can contribute to degrade the gelatin coating of the transwell inserts, facilitating MSC migration. Interestingly, MMP-9 is also able to cleave osteopontin into fragments with different biological activity, some of which particularly prone in the promotion of cell migration and invasion, as demonstrated *in vitro* for hepatocellular carcinoma cells^51^. Although these were amongst the most represented molecules in our screening, we cannot rule out that other chemotactic mediators contained in EV could be responsible for the increased MSC migration. Further clarifying this would require knock-down experiments evaluating the molecule or combination of molecules without which migration in response to DC-EV could no longer be observed. The DC-derived EV population enriched in exosomes constitutes nanosized carriers, likely containing several chemotactic mediators, some of which able to interact with each other in order to promote enhanced cell migration. This supports a local role for EV in promoting healing, which could be manipulated both by modulating the cargo and secretion of EV derived from the immune cells, or by the delivery of exogenous EV.

In the context of future new regenerative therapies development, we here demonstrate the capacity of EV from naïve DC to promote MSC migration, which could be exploited for the local recruitment of either endogenous or transplanted cells towards injury sites. Also, the potential for EV from different cell populations to lead MSC to perform different functions, should be explored. This could support time-controlled changes to the injury microenvironment, that can enhance the regenerative response. Further studies addressing the use of EV in promoting tissue repair are granted and necessary to explore their full potential.

## Methods

### Ethical approval

Human biological samples used in this work and all the procedures followed to obtain them were conducted according to the principles of the Declaration of Helzinki. Surplus buffy coats for monocytes isolation were from healthy blood donors and donated by Immunohemotherapy Department of Centro Hospitalar de São João (CHSJ, Porto, Portugal). MSC were isolated from discarded human bone marrow tissues of patients undergoing total hip arthroplasty^18^, and obtained from the Orthopaedics Department of CHSJ. All blood donors and patients gave informed written consent. Experimental protocols were approved by CHSJ Ethics Committee for Health. No information on age, sex, or any other identifying element was provided to the researchers, so all samples were analysed anonymously.

### MSC isolation and culture

MSC were isolated from bone marrow tissue of femur or tibia of orthopaedic patients as previously reported^18^. MSC identity was confirmed according to the criteria defined by the International Society for Cellular Therapy^52^. For each experiment, MSC were cultured in MSC growth medium - low glucose Dulbecco’s modified Eagle’s medium (DMEM) + Glutamax (Invitrogen), supplemented with 10% heat inactivated MSC-compatible Foetal Bovine Serum (FBS, HyClone™ or Gibco™) and 1% P/S. Cells were maintained in a humidified incubator, at 37 °C and 5% CO_2_. MSC from two different donors were used, from passage 5 to 9.

### DC differentiation and EV production

Monocytes were isolated from buffy coats of healthy blood donors using RosetteSep™ human monocyte enrichment antibody cocktail (STEMCELL Technologies), as previously described^53^. DC cultures were set up with 60–100 × 10^6^ human peripheral blood monocytes, plated at 20 × 10^6^ cells per 100 mm diameter petri dishes, in DC differentiating media - RPMI-1640 + Glutamax (Invitrogen) cell culture media, with 10% heat inactivated FBS (Lonza) and 1% penicillin G-streptomycin (P/S; Invitrogen), further supplemented with 50 ng/mL IL-4 and GM-CSF (Immunotools). After 4 days of differentiation, cells were washed with PBS and re-cultured for 3 additional days in EV producing media. This media was obtained from ultracentrifuged (100,000 × g, for 2 h at 4 °C) RPMI supplemented with 10% FBS, which was then diluted to 1% FBS, and further supplemented with 1% P/S, IL-4 and GM-CSF, as above. Absence of bovine-EV in the ultracentrifuged RPMI + 10% FBS media was confirmed by transmission electron microscopy (Supplementary Fig. S3). Cells were maintained in a humidified incubator, at 37 °C and 5% CO_2_. DC cultures in EV-producing media were monitored by optical microscopy and no changes in cell morphology or general culture condition were observed (Supplementary Fig. S4).

### EV isolation

DC-derived EV were isolated from cell culture media by differential centrifugation: cell culture media was collected and centrifuged at 2,000 × g, for 20 min, at 4 °C; the supernatant was recovered and centrifuged again, now at 10,000 × g, for 20 min, at 4 °C; the supernatant was again recovered and ultracentrifuged, at 100,000 × g, for 2 h, at 4 °C, in a Beckman Optima L80-XP ultracentrifuge. EV pellets were resuspended in PBS and supernatant (100 K supernatant; UC control) collected, and stored at −80 °C. A similar volume of EV producing media, that was never in contact with cells, was also ultracentrifuged as described above. Protein pellets from ultracentrifugation were resuspended in PBS and stored at −80 °C, as for DC-EV. For each EV or control sample, protein was quantified directly using *DC*™ Protein Assay (Bio-Rad), and the calculated protein concentration was used as a measure for functional assays.

### EV morphology and size characterization

EV morphology was analysed by transmission electron microscopy (TEM). DC-derived EV were adsorbed onto 400-mesh formvar nickel grids and then negatively stained with uranyl acetate 5% for 30 sec. Samples were imaged in a Jeol JEM 1400 electron microscope.

For size and concentration characterization of EV suspensions, 10 μg of DC-derived EV by nanoparticle tracking analysis (NTA) in a NanoSight NS300 with NTA3.0 software. Size distribution and EV suspension polydispersity was further characterized by dynamic light scattering (DLS) in a Zetasizer Nano ZS device.

### Flow cytometry analysis of EV

For flow cytometry analysis, EV were first coupled to 4 μm size aldehyde/sulphate latex beads 4%(w/v) (Invitrogen). Sonicated beads were incubated with DC-derived EV at 1:1 ratio (vol:protein quantity), first for 1 h at room temperature, and then overnight at 4 °C, under mild agitation. Protein pellets from cell-free EV producing media processed as above or 100 K supernatant were adsorbed to beads for negative controls. Functional groups remaining on the beads were blocked by incubation with glycine 100 mM for 30 min at room temperature, under mild agitation. EV were immunostained with anti-human CD63 PE-conjugated, or anti-human HLA-DR FITC-conjugated antibodies (all from Immunotools), and then fixed with paraformaldehyde 4%(w/v). For MMP9 detection, bead-coupled EV were fixed, permeabilized (Triton X-100 0.2%(v/v), 5 min), and incubated with primary antibody (Abcam), followed by Cy3-conjugated secondary antibody. Immunostaining with matching isotypic antibodies were used as control. Samples were analysed on a FACS Calibur flow cytometer (BD Biosciences) or ImageStream®^X^ imaging flow cytometer (Amnis, EMD Millipore). Beads with coupled EV were gated according to forward and side scatter parameters with BD CellQuest software (BD Biosciences) or Inspire software (Amnis, EMD Millipore), respectively, and 20,000 events were recorded. Data was analysed using FlowJo Software (TreeStar, Inc) or IDEAS software (Amnis, EMD Millipore), respectively.

### Western blotting

Protein content of DC lysates, EV pellets and 100 K supernatants was quantified as above and the same amount of protein (7 μg) prepared in non-reducing (for CD63) or reducing (for calnexin and osteopontin) conditions. Samples were denaturated at 65 °C for 15 min, resolved on SDS-PAGE 10% polyacrylamide gels (Bio-Rad), and then transferred to nitrocellulose membranes. Membranes were blocked and probed overnight with the primary antibody anti-human CD63 (BD Pharmingen™), anti-human calnexin (Abcam) or anti-human osteopontin (Rockland), followed by HRP-conjugated secondary antibody (GE Healthcare). Membranes were incubated with ECL substrate and chemiluminescent signal detected upon exposure to autoradiographic films (all from GE Healthcare). Full scans of the films are available in Supplementary Fig. S5.

### EV internalization assay

DC-derived EV were labelled with PKH26 0.5 μM dye (Sigma-Aldrich), for 5 min, at 37 °C, and free dye washed in VivaSpin**®** centrifugal columns (10 kDa cut-off). Labelled EV were co-cultured with MSC (1.5 × 10^3^ cell/mL). Pellets of cell-free EV producing media ultracentrifuged as above, and PBS were labelled with PKH26 and used as negative controls. At different timepoints, cells were fixed with paraformaldehyde 2%(w/v), stained with FITC-conjugated phalloidin (Life Technologies), and mounted on slides with fluoroshield with DAPI. Images were acquired by laser scanning confocal microscopy (Leica TCS SP2) using the same settings for all conditions tested, and Z-stack images analysed using Fiji software. For internalization quantification, cells were defined as ROI according to phalloidin staining (green channel) and then fluorescence intensity PKH26 (red channel) determined on Fiji software.

### MSC proliferation assays

MSC (20 × 10^3^ cells/mL) cultured in EV-free media were stimulated for 24 h with 50 µg of indicated stimuli. MSC were then immunostained for Ki67 (ThermoFisher Scientific), followed by fluorochrome-conjugated secondary antibody, and mounted with fluoroshield with DAPI. Secondary antibody alone was used as negative control. Images from 20 random locations were acquired and analysed using Fiji software for number of nuclei and Ki67^+^ cells counting.

Cell proliferation over time was assessed indirectly with resazurin cell viability assay according to manufacturer’s protocol. Briefly, MSC (1.5 × 10^4^ cells/mL) cultured in EV-free media were stimulated with 10 µg DC-derived EV or appropriate controls, and at each timepoint, incubated with resazurin (10 µg/mL, 4 h, 37 °C). Media fluorescence was measured at 590 nm (Synergy HT plate reader (Biotek)).

### MSC differentiation assays

MSC (4 × 10^4^ cells/mL) cultured in EV-free media were stimulated as indicated for 6 h (gene expression), or until day 14 (ALP activity staining). Basal media was supplemented with ultracentrifuged FBS, and 10 µg of stimuli were added as indicated. Osteogenesis inducing media was further supplemented with Dexamethasone 10^−7^ M, β-glycerophosphate 10^−2^ M, ascorbic acid 5 × 10^−5^ M (all from Sigma-Aldrich). For gene expression analysis, MSC RNA was extracted with TRIzol® (Invitrogen) following manufacturer’s protocol. RNA concentration was determined using a NanoDrop ND-1000 device (ThermoFisher Scientific), and its integrity confirmed by agarose gel electrophoresis. RNA (2 µg) was then digested with TURBO DNA-free Kit (Life Technologies) according to manufacturer’s instructions, and used for cDNA synthesis using random hexamers and SuperScript® III Reverse Transcriptase (all from Life Technologies). RT-qPCR was performed in a iQ5 Real-Time PCR Detection System (Bio-Rad) using cDNA, gene specific primers and iQ SYBR Green Supermix (Bio-Rad). Gene expression was considered for Cq threshold cycles <35. GAPDH was used as reference gene and relative gene expression was calculated by the ΔC_q_ method (according to MIQE guidelines). For ALP activity staining, MSC were fixed with paraformaldehyde 4% (w/v) and incubated with a Fast Violet B salt solution (0.25 mg/mL) containing Naphthol AS-MX phosphatase alkaline solution (diluted 1:25) (all from Sigma-Aldrich), for 45 min at room temperature. Cells were washed and imaged on an Olympus CKX41 microscope.

### Transwell migration assay

Transwell assays were set up in 24-well plates using inserts with membranes of 8 µm pore size (BD Falcon™). Insert membranes coated with gelatin (1% (w/v), 1 h at 37 °C) were seeded with 0.2 × 10^6^ MSC (TC, top compartment) and indicated stimuli were placed on the bottom wells for experiments with gradient (BC, bottom compartment), using serum-free DMEM. For gradient disruption control, 50 μg of EV were diluted in serum-free DMEM and media equally distributed by the top and bottom compartments. DC total conditioned media and protein pellets from cell-free EV producing media, processed as above, were used as controls. After 8 h, insert membranes were fixed with paraformaldehyde 4% (w/v), non-migrated cells removed with cotton swabs, membranes cut from the inserts and mounted using fluoroshield with DAPI. Migrated cells were counted in the entire insert membranes using an epifluorescence microscope, and migration index calculated for normalization across experiments as the ratio of migrated MSC in the test condition to the MSC migrated in the respective control condition, serum free media.

### 2D chemotaxis assay

Chemotaxis assays were performed with Collagen IV-coated μ-Slide Chemotaxis microfluidics devices (ibidi® GmbH), as recommended by the manufacturer. Briefly, 1 × 10^4^ MSC in EV-free media were seeded in the central channel of the chemotaxis chamber and allowed to adhere for 1 h. Afterwards, a chemotactic gradient was established by addition of 10 μg of indicated stimuli to the right side of the chamber. Cell movement in the central channel was recorded by time-lapse video microscopy (see Supplementary Video S1) in an IN Cell Analyzer 2000 (GE Healthcare), using a Nikon 10x/0.45 NA Plan Fluor objective, with controlled temperature and CO_2_. Brightfield images were acquired every 10 min during 13 h, and processed using IN Cell Investigator software (GE Healthcare). Movement of 20 cells per condition was tracked by image analysis with MTrackJ plugin (Supplementary Fig. 1), and chemotaxis parameters determined using the Chemotaxis Tool plugin (both for Fiji software^54^).

### EV chemokines content analysis

DC-derived EV pellets or total conditioned media of 3 different DC donors were pooled (1:1:1) to 60 µg of protein, followed by hybridization on a semi-quantitative RayBio® C-Series human cytokine antibody array (RayBiotech), following manufacturer’s protocol. Full membrane scans are available in Supplementary Fig. 2. IL-6 and MCP-1 were validated by ELISA assay in DC-derived EV pellets and 100 K supernatant pooled as above. Standard ABTS ELISA development kit (PeproTech) was used, following manufacturer’s protocol, and cytokine concentration was determined by comparison with a standard curve.

### Gelatin zymography

Gelatinase activity of MMPs was analysed on conditioned media collected separately from TC and BC compartments of transwell assay, and processed as described before^17^, or directly on EV pellets and 100 K supernatants. Where indicated, EV and 100 K supernatant samples were incubated with proteinase K (10 μg/mL, 15 min, 37 °C, Sigma-Aldrich), for soluble proteins digestion, before or after EV lysis. Protease was heat-inactivated (60 °C, 10 min) in the presence of phenylmethylsulfonyl fluoride 5 mM. Protein was quantified and the same amount (4 μg) resolved in gelatin-containing zymography gels as previously described^17^, followed by gel incubation overnight with MMP substrate buffer (CaCl_2_ 10 mM in Tris 50 mM buffer, pH7.5). Gels were stained with Coomassie Brilliant Blue R-250 (Sigma-Aldrich), washed until clear bands were visible, and imaged in a calibrated GS-800 densitometer (gel full scans are available in Supplementary Fig. S6).

### Statistical analysis

Statistical analysis was performed on Prism v6.0c software. Gaussian distribution was tested by D’Agostino-Pearson omnibus normality test. For non-normal data, Kruskal-Wallis followed by Dunn’s multiple comparison test was used. For DC-EV internalization assay, resazurin, and chemotaxis assays, 2-way ANOVA followed by multiple comparisons testing was used. Statistical significance was considered for p-value < 0.05.

## Electronic supplementary material

Supplementary information

MSC tracking on images acquired by time-lapse video microscopy (Related to Figure 4).
