# Supplementary material for: Dendritic Cell-derived Extracellular Vesicles mediate Mesenchymal Stem/Stromal Cell recruitment
Source: Sci Rep. 2017 May 10;7:1667. doi: 10.1038/s41598-017-01809-x (PMC5431789; doi:10.1038/s41598-017-01809-x)
Supplement: Supplementary file 1 — Supplementary information [file 41598_2017_1809_MOESM1_ESM.pdf]

Supplementary Information

Dendritic Cell-derived Extracellular Vesicles mediate Mesenchymal Stem/Stromal Cell recruitment

Andreia M. Silva, Maria I. Almeida, José H. Teixeira, André F. Maia, George A. Calin, Mário A. Barbosa, Susana G. Santos

Supplementary Figure S1

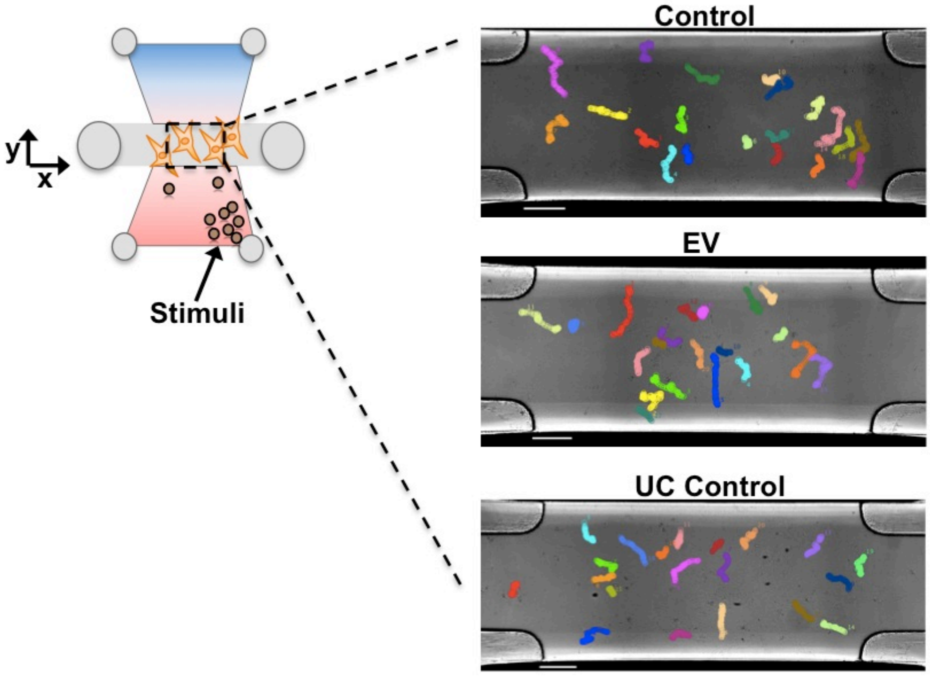

Supplementary Figure S1. MSC tracking in the chemotaxis devices (Related to Figure 4). Exemplificative images of the pathways manually tracked for MSC seeded in the central channel of the chemotaxis microfluidics devices, for unstimulated control condition and upon EV and 100K ultracentrifugation supernatant gradient formation. Scale bar: 200µm.

Supplementary Figure S2

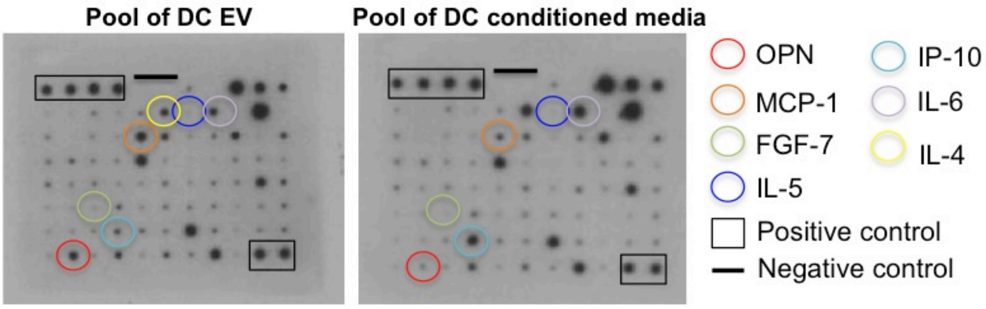

Supplementary Figure S2. Characterization of DC exosomes protein content by cytokine antibody array hybridization (Related to Figure 5). Scans of membranes hybridized with DC-EV pellets and conditioned media pools. Spots corresponding to selected chemoattractants with the highest absolute fold-change are highlighted, along with membrane positive and negative internal controls.

Supplementary Figure S3

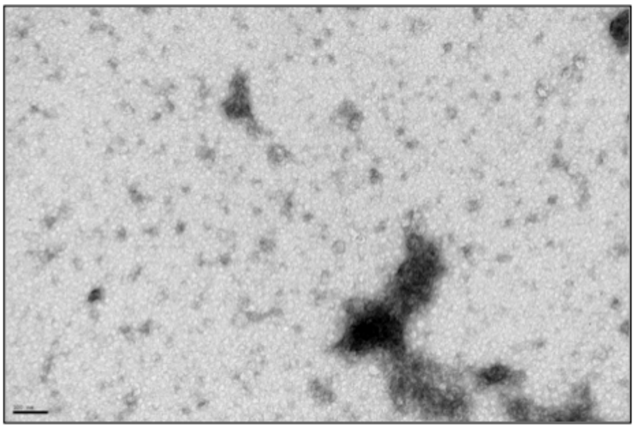

Supplementary Figure S3. Transmission electron microscopy analysis of ultracentrifuged RPMI+10% FBS media used for DC cultures. Transmission electron microscopy micrograph of EV-producing media used for DC culture, showing dark protein agglomerates but not EV of bovine origin. Scale bar: 100 nm.

Supplementary Figure S4

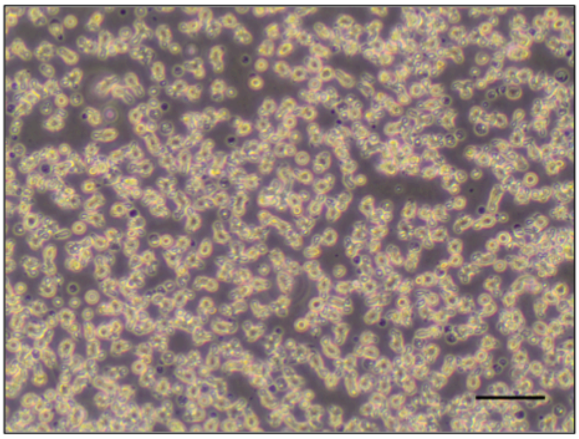

Supplementary Figure S4. Overall aspect of DC after 3 days in culture in ultracentrifuged RMPI+1% FBS. DC maintain their integrity with reduced cell death in the decreased serum conditions used. Scale bar: 50 μm.

Supplementary Figure S5

Main Fig. 1d

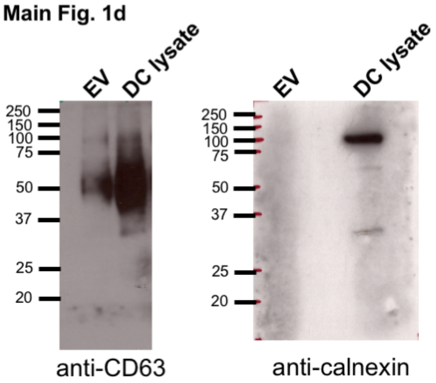

Main Fig. 5b

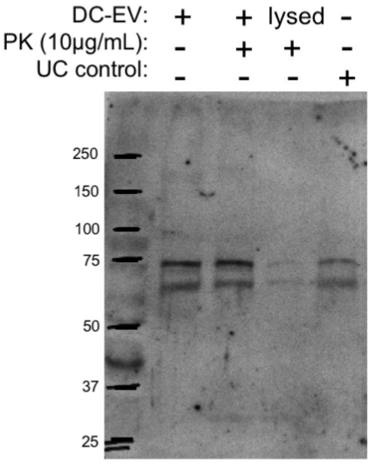

Supplementary Figure S5. Full-length scan of Western blots. Correspondence with cropped figures is indicated.

Supplementary Figure S6

Main Fig. 6a

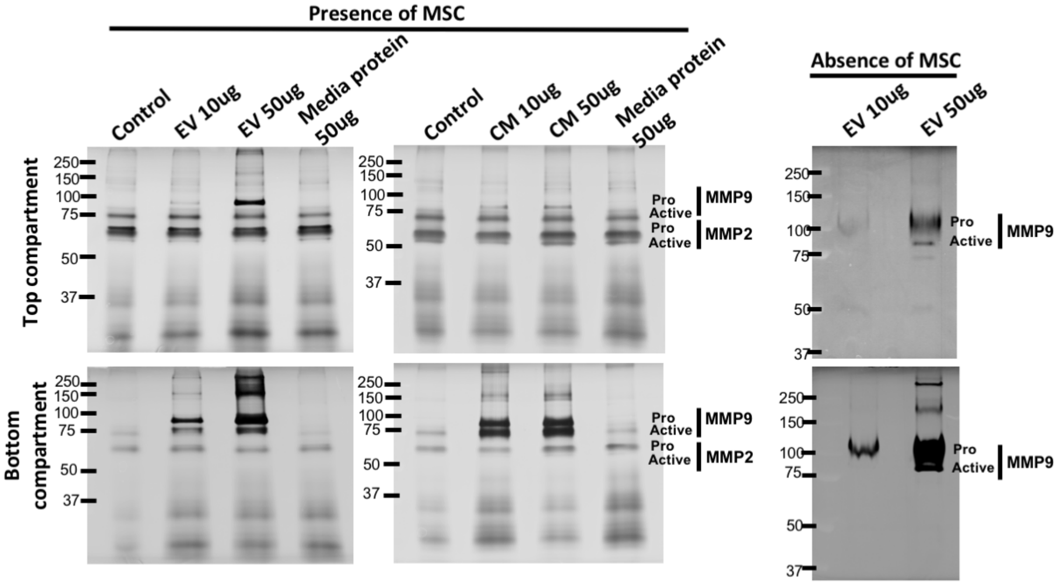

Main Fig. 6C

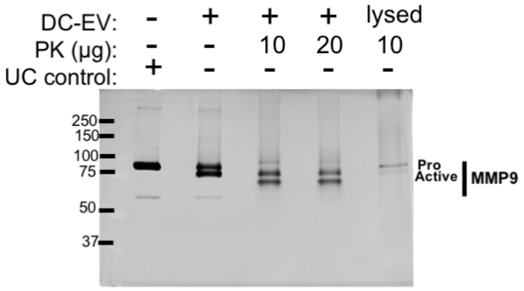

Supplementary Figure S6. Full-length scan of gelatin zymography gels. Correspondence with cropped figures is indicated.

Supplementary Video S1. MSC tracking on images acquired by time-lapse video microscopy (Related to Figure 4). An exemplificative time-lapse video is shown for MSC exposed to DC-derived EV gradient. Migrating MSC were tracked manually using the MTrackJ plugin for Fiji, and tracked pathways are shown in colour along time.
